# Parvalbumin-Deficiency Accelerates the Age-Dependent ROS Production in Pvalb Neurons *in vivo*: Link to Neurodevelopmental Disorders

**DOI:** 10.3389/fncel.2020.571216

**Published:** 2020-09-28

**Authors:** Lucia Janickova, Beat Schwaller

**Affiliations:** Department of Neurosciences amd Movement Science, Section of Medicine, University of Fribourg, Fribourg, Switzerland

**Keywords:** parvalbumin, autism, schizophrenia, ROS, mitochondria

## Abstract

In neurodevelopmental disorders (NDDs) including autism spectrum disorder (ASD) and schizophrenia, impairment/malfunctioning of a subpopulation of interneurons expressing the calcium-binding protein parvalbumin (PV) –here termed Pvalb neurons– has gradually emerged as a possible cause. These neurons may represent a hub or point-of-convergence in the etiology of NDD. Increased oxidative stress associated with mitochondria impairment in Pvalb neurons is discussed as an essential step in schizophrenia etiology. Since PV downregulation is a common finding in ASD and schizophrenia individuals and PV-deficient (PV−/−) mice show a strong ASD-like behavior phenotype, we investigated the putative link between PV expression, alterations in mitochondria and oxidative stress. In a longitudinal study with 1, 3, and 6-months old PV−/− and wild type mice, oxidative stress was investigated in 9 Pvalb neuron subpopulations in the hippocampus, striatum, somatosensory cortex, medial prefrontal cortex, thalamic reticular nucleus (TRN) and cerebellum. In Pvalb neuron somata in the striatum and TRN, we additionally determined mitochondria volume and distribution at these three time points. In all Pvalb neuron subpopulations, we observed an age-dependent increase in oxidative stress and the increase strongly correlated with PV expression levels, but not with mitochondria density in these Pvalb neurons. Moreover, oxidative stress was elevated in Pvalb neurons of PV−/− mice and the magnitude of the effect was again correlated with PV expression levels in the corresponding wild type Pvalb neuron subpopulations. The PV-dependent effect was insignificant at 1 month and relative differences between WT and PV−/− Pvalb neurons were largest at 3 months. Besides the increase in mitochondria volume in PV’s absence in TRN and striatal PV−/− Pvalb neurons fully present already at 1 month, we observed a redistribution of mitochondria from the perinuclear region toward the plasma membrane at all time points. We suggest that in absence of PV, slow Ca^2+^ buffering normally exerted by PV is compensated by a (mal)adaptive, mostly sub-plasmalemmal increase in mitochondria resulting in increased oxidative stress observed in 3- and 6-months old mice. Since PV−/− mice display core ASD-like symptoms already at 1 month, oxidative stress in Pvalb neurons is not a likely cause for their ASD-related behavior observed at this age.

## Introduction

The etiology of neurodevelopmental disorders (NDDs) including autism spectrum disorder (ASD), schizophrenia and attention deficit hyperactivity disorder remains unclear. Although each NDD is characterized by a specific trajectory with respect to onset, symptoms or behavioral alterations, as well as brain regions and cell types implicated in this process, they share genetic etiology (Schork et al., 2019) and also disorder-associated comorbidities. This hints toward impairments of similar and/or convergent pathways in the various NDD. Such a point-of-convergence may exist in the malfunctioning/impairment of GABAergic interneurons (Marin, 2012; Schork et al., 2019) mostly in the ones expressing the calcium-binding protein parvalbumin (PV) (Ferguson and Gao, 2018) hereafter called Pvalb neurons. This interneuron type present, e.g., in cortex, hippocampus, striatum and cerebellum of rodents and humans (for details, see, Celio, 1990; del Rio et al., 1994; Hashemi et al., 2017; Soghomonian et al., 2017) is characterized by fast, non-adaptive firing and rapid AP kinetics. Pvalb neurons target the perikarya or axon initial segments of excitatory (and inhibitory) neurons and form strong autapses (Deleuze et al., 2019), which make them particularly suitable for enabling and controlling synchronization of neuron ensembles (Sohal et al., 2009; Bohannon and Hablitz, 2018) resulting in oscillatory activity. At the morphological level, a majority of Pvalb neurons is surrounded by perineuronal nets (PNN) that allow for their identification irrespective of PV expression levels as shown before (Filice et al., 2016).

In schizophrenia, a decrease in the number of PV-immunoreactive (PV^+^) neurons resulting from PV downregulation (initially presumed to result from Pvalb neuron loss) is observed in postmortem brains of affected individuals, as well as in mouse models of schizophrenia (Do et al., 2009; Powell et al., 2012). In most cases, a concomitant decrease in GAD67 (*GAD1*) occurs indicative of a common/similar regulation of the *PVALB* and *GAD1* genes. The decrease of PV and GAD67 is considered as a downstream effect of NMDA receptor (NMDAR) hypofunction in pyramidal cells resulting in decreased activity of Pvalb neurons (Gonzalez-Burgos and Lewis, 2012; Gonzalez-Burgos et al., 2015) proposed to reduce network gamma oscillatory activity (Volman et al., 2011). An extensively studied hypothesis is centered on oxidative stress-mediated Pvalb neuron impairment likely associated with mitochondrial dysfunction (Steullet et al., 2017), as was also proposed for the etiology of ASD (Bader et al., 2011; Tang et al., 2013). In the redox dysregulation model of schizophrenia oxidative stress is viewed as the “integrator” leading to Pvalb neuron impairment (Steullet et al., 2017). Accordingly, genetic and/or environmental factors weaken antioxidant defense systems leading to NMDAR hypofunction and PV downregulation (reviewed in Hardingham and Do, 2016). However, during hippocampal maturation *in vitro*, NMDAR inhibition and oxidative stress differentially alter PV expression and gamma oscillation activity (Hasam-Henderson et al., 2018) indicating that general oxidative stress cannot be the main mechanism underlying the decrease in PV expression and that it cannot mechanistically explain the effects of NMDAR hypofunction (Hasam-Henderson et al., 2018).

In the case of ASD, GABA system dysfunction including impaired Pvalb neuron function is a well-accepted hypothesis (Chattopadhyaya and Cristo, 2012; Coghlan et al., 2012; Marin, 2012). Yet, the putative role of the protein PV lending its name to the Pvalb neuron subpopulation has been investigated to a much lesser extent in ASD (and schizophrenia). Reduced numbers of PV^+^ neurons in ASD patients and in animal models of ASD were initially presumed to be the result of a lower number of Pvalb neurons (Gogolla et al., 2009), reviewed in Schwaller (2020). However, in many cases the observed decreased number of PV^+^ neurons –at least in mice– is the result of PV downregulation, i.e., in low PV-expressing neurons PV expression levels fall below the detection threshold [e.g., in Shank1−/−, Shank3B−/− (Filice et al., 2016), Cntnap2−/− (Lauber et al., 2018), and VPA mice (Lauber et al., 2016)]. In line, the most strongly downregulated transcript in cerebral cortex of ASD patients is *PVALB* mRNA (Parikshak et al., 2016) and several transcripts of genes related to synaptic transmission and mitochondria (Schwede et al., 2018). Transcriptomic network analysis of three mouse ASD and schizophrenia models identified four modules [M; two cortical (c) and two hippocampal (h)] of co-expressed genes dysregulated in all three animal models (Gordon et al., 2019). The upregulated cM1 module is enriched for genes implicated in ‘morphogenesis of branching structures,’ while the downregulated cM2 is annotated as mitochondrial related energy balance (‘energy-coupled proton transport’ and ‘respiratory electron transport chain’). Importantly, Expression Weighted Cell-Type Enrichment (EWCE) analysis uncovered a significant increase in cM2 (mitochondrial) genes in fast-firing inhibitory neurons, presumably Pvalb neurons.

We have previously shown that a reduction or complete elimination of PV in PV + /− and PV−/− mice results in a robust ASD-like behavior phenotype showing all ASD core symptoms, as well as ASD-associated comorbidities (Wöhr et al., 2015). Moreover, in all systems investigated so far [fast-twitch muscle, PV-overexpressing epithelial (MDCK), oligodendrocyte-like (CG4) cells (for details, see, Schwaller, 2020)] and Pvalb neurons *in vivo* (Janickova et al., 2020), PV downregulation leads to a compensatory/homeostatic upregulation of mitochondria volume (Schwaller, 2020), while ectopic PV expression in all neurons of Thy-PV transgenic mice decreases the mitochondria volume evidenced in the striatum (Maetzler et al., 2004). In distinct Pvalb neuron populations in adult PV−/− mice, the mitochondrial volume is augmented and the relative increase strongly correlates with the PV concentration prevailing in the various WT Pvalb neuron subpopulations in the different brain regions, i.e., the higher the concentration of PV, the higher the PV loss-induced increase in mitochondria volume (Janickova et al., 2020). Interestingly, both in CG4 cells and more relevant in PV−/− Pvalb neurons, the increase in mitochondria volume and dendritic mitochondria length is associated with increased branching and length of dendrites (Lichvarova et al., 2019; Janickova et al., 2020), in full agreement with the reported increase in genes of module cM1 (‘morphogenesis of branching structures’) in three ASD and schizophrenia mouse models (Gordon et al., 2019). Thus, we set out to investigate the timeline of events caused by the absence of PV. More precisely, we analyzed the trajectory (temporal changes) in mitochondria volume and mitochondria distribution in selected Pvalb neurons and the level of oxidative stress in the same Pvalb neuron subpopulations, where we had had previously observed an increase in mitochondria volume in adult (3–5 months) PV−/− mice (Janickova et al., 2020). We chose three time points: 1, 3, and 6 months. The first one, since mice deficient for PV (PV + /−, PV−/−) show ASD core symptoms –reduced communication and social interaction and repetitive/stereotyped behavior– already at PND25-30, as well as changes in the morphology (dendrites) of striatal Pvalb neurons (Wöhr et al., 2015). At 3 months, some ASD-like behavior persists in PV−/− mice (Wöhr et al., 2015) and moreover, clear increases in mitochondria volume occur in 3–5 months-old PV−/− Pvalb neurons (Janickova et al., 2020). This is also the time point, when increased oxidative stress (and PV downregulation) is observed in mouse schizophrenia models (Steullet et al., 2018; Cabungcal et al., 2019). Our results indicate that the ASD-like phenotype in PV−/− mice emerges before the onset of an increase in oxidative stress hinting toward absence/reduction of PV as the main contributor to the behavioral ASD-like phenotype.

## Results

### Age-Dependent Increase in Oxidative Stress in WT Pvalb Neurons Evidenced by 8-Oxo-dG Staining

**FIGURE 1:**
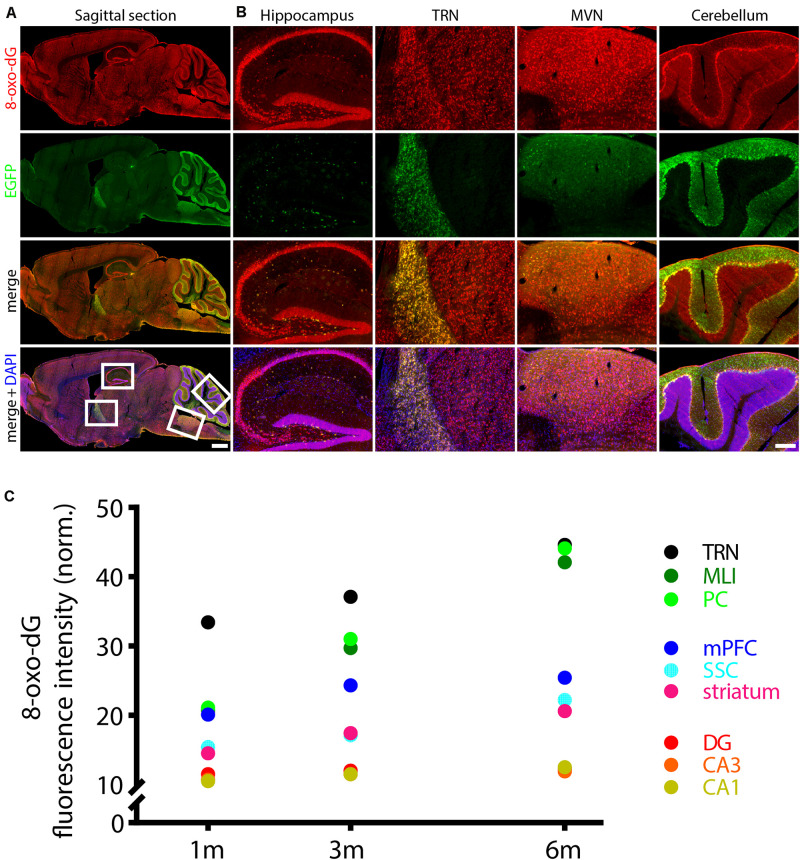
Age-dependent increase in oxidative stress in WT Pvalb neurons evidenced by 8-oxo-dG staining. **(A)** Sagittal brain section from a 3-month old WT mouse stained for 8-oxo-dG (oxidative stress marker, red), EGFP (Pvalb neurons expressing EGFP, green) and DAPI (nuclei, blue). Regions with strong 8-oxo-dG staining (white boxes in low-left image in **A**) –hippocampus, TRN, MVN and cerebellum– are shown at higher magnification in **(B)**. Note the higher densities of EGFP^+^ Pvalb neurons and/or neuropil (fibers) in these regions resulting in partial yellow staining in the merged images. Scale bar in **(A)** 1 mm, in **(B)** 200 μm. Sagittal brain sections scanned by the NanoZoomer serve for demonstration purposes and were not analyzed in the study. **(C)** Quantitative analyses were performed on confocal microscopy images. Brain sections were scanned in nine regions – 3 hippocampal regions (CA1, CA3, and DG), striatum, SSC, mPFC, 2 cerebellar regions [Purkinje cell layer (PC) and in molecular layer interneurons (MLI)] and TRN. Fluorescence signal quantifications were carried out in 1, 3, and 6-months old mice [normalized values (mean ± SD) are reported in Table 1]; for clarity, error bars and regression lines are omitted; R^2^ values are from linear regression analyses using the three time points: 1, 3, and 6 months and are listed in Table 1.

The strong activity of Pvalb neurons (fast, high-frequency firing) is coupled to elevated metabolism, high mitochondrial activity and associated ROS production. Subsequently, Pvalb neurons contain substantially more mitochondria in their somata, dendrites and axons than any other interneuron subpopulation or pyramidal cells (reviewed in Kann et al., 2014) and are thus highly susceptible to oxidative stress (Kann, 2016). Resulting from the high mitochondria density, the outline of Pvalb cells can be often recognized on brain sections simply by a general staining for mitochondria, particularly in Pvalb neurons of PV−/− mice (Janickova et al., 2020); examples in TRN are shown in Supplementary Figure 1. In this study, we made use of two transgenic mouse lines, where EGFP is selectively expressed in Pvalb neurons (Orduz et al., 2013). In the control (WT) line, mice have two functional *Pvalb* alleles, while the other one is null-mutant (PV−/− or KO) for *Pvalb*. Since the transgene driving EGFP expression is not associated with the endogenous *Pvalb* gene locus, EGFP expression is not coupled to endogenous PV expression. That is, EGFP expression is indistinguishable in WT and KO mice (Janickova et al., 2020). Oxidative stress was determined by immunofluorescence detection of the DNA oxidation product 8-oxo-dG, globally at low magnification on sagittal sections and moreover, in the same Pvalb neuron subpopulations, where we had previously investigated the effect of PV on mitochondria volume and cell morphology, i.e., in the somatosensory and medial prefrontal cortex (SSC, mPFC), striatum, thalamic reticular nucleus (TRN), hippocampal regions DG, CA3 and CA1 and cerebellum [Purkinje cells and molecular layer interneurons (MLI)]. On sagittal sections of 3-months old WT mice scanned by the NanoZoomer at low magnification (Figure 1A), several brain regions showed rather strong 8-oxo-dG staining: higher magnification images of the hippocampal DG granule cell layer and the CA3–CA1 pyramidal cell layer, the TRN, the medial vestibular nucleus (MVN) and the cerebellum, in particular the Purkinje cell layer and MLI are shown in Figure 1B. In the MVN, staining was mostly confined to fibers, but was also observed in some EGFP^+^ Pvalb neuron somata present in the parvocellular part of the MVN (Supplementary Figure 2) as reported before in rat (Puyal et al., 2002). Essentially all regions showing strong 8-oxo-dG staining also contained higher densities of EGFP^+^ Pvalb neurons and/or neuropil (fibers) resulting in partial yellow staining in the merged images (Figures 1A,B). In Pvalb neuron populations of WT mice analyzed by confocal microscopy, 8-oxo-dG fluorescence signals (in arbitrary units) were first normalized, taking into consideration the different sizes and morphologies of Pvalb neurons as described before (Janickova et al., 2020). We observed a near-linear age-dependent increase in 8-oxo-dG signals in mice of 1, 3, and 6 months (Figure 1C and Table 1), most evident in the group of high PV-expressing neurons (TRN, MLI, and PC). Linear regression analyses (R^2^) revealed high linearity values ≥ 0.97 for medium-to-high PV-expressing Pvalb neurons including TRN, MLI, PC, SSC, and striatum (Figure 1C and Table 1). Of note, 8-oxo-dG signals in TRN Pvalb neurons at 1 month were already considerably higher than in all other Pvalb neuron subpopulations [ANOVA: *F*(8,36) = 64.63; *p* < 0.0001; Tukey’s multiple comparison test: *p* < 0.0001 for all other Pvalb subpopulations], yet the time-dependent increase (slope) was not much higher than for medium PV-expressing Pvalb neurons (striatum, SSC) and clearly smaller than for MLI and PC that showed the strongest time-dependent increase. An exception was the subpopulation of mPFC Pvalb neurons (*R*^2^ = 0.82), where 8-oxo-dG signals at 6 months were only marginally higher than at 3 months. In low PV-expressing hippocampal (DG, CA3, CA1) neurons, 8-oxo-dG signal intensities in young (1 month) mice were the lowest of all tested Pvalb neuron subpopulations, and the relative age-dependent increase was marginal. Thus, both parameters (WT fluorescence intensity at 3 months, slope) generally correlated positively with the PV concentration previously estimated in the various Pvalb neuron populations [see Figure 1E in Janickova et al. (2020) and Supplementary Figure 4B]. Of note, these changes were not correlated with the relative densities of the mitochondria in the soma cytoplasm of different Pvalb neuron populations, which are rather constant in all Pvalb neurons: 7.8 ± 1.5 vol./vol.% (Janickova et al., 2020).

**TABLE 1:** Quantitative results and statistical analyses of 8-oxo-dG and DHE staining intensities are shown as mean ± standard deviations in all investigated brain regions.

8-oxo-dG		1 month		3 months		6 months								
			
		WT	KO	WT	KO	WT	KO							WT

		Mean ± SD	Mean ± SD	Mean ± SD	Mean ± SD	Mean ± SD	Mean ± SD	Effect of age	→	*p*-value	Effect of genotype	→	*p*-value	*R*^2–^value
	**DG**	10.17 ± 1.36	10.51 ± 1.46	10.60 ± 0.97	10.77 ± 0.97	10.85 ± 0.88	11.11 ± 0.85	*F*(2,24) = 0.8305		0.4480	*F*(1,24) = 0.3980		0.5341	0.9306
	**CA3**	12.04 ± 0.93	12.23 ± 1.21	12.91 ± 0.81	12.91 ± 0.88	13.40 ± 0.65	13.98 ± 0.41	*F*(2,24) = 8.3030		0.0018	*F*(1,24) = 0.6919		0.4137	0.9263
	**CA1**	10.60 ± 1.27	11.47 ± 1.44	11.55 ± 0.71	11.81 ± 0.89	12.59 ± 0.56	12.67 ± 1.03	*F*(2,24) = 6.0060		0.0077	*F*(1,24) = 1.1330		0.2977	0.9921
	**Striatum**	14.46 ± 0.97	15.36 ± 1.71	17.41 ± 1.81	20.89 ± 1.71	20.54 ± 1.75	21.88 ± 1.33	*F*(2,24) = 41.2000		<0.0001	*F*(1,24) = 10.8800		0.0030	0.9905
	**SSC**	15.35 ± 2.32	16.41 ± 1.27	17.06 ± 1.53	20.82 ± 2.25	22.20 ± 1.18	23.67 ± 2.08	*F*(2,24) = 34.0800		<0.0001	*F*(1,24) = 8.94900		0.0063	0.9725
	**mPFC**	20.09 ± 1.45	20.87 ± 1.87	24.24 ± 3.09	30.82 ± 3.60	25.37 ± 3.10	32.99 ± 3.54	*F*(2,24) = 25.4600		<0.0001	*F*(1,24) = 22.2800		<0.0001	0.8232
	**PC**	21.05 ± 2.47	22.62 ± 1.61	30.99 ± 4.88	41.22 ± 2.82	44.01 ± 4.53	49.48 ± 2.24	*F*(2,24) = 141.600		<0.0001	*F*(1,24) = 22.5200		<0.0001	0.9986
	**MLI**	20.68 ± 2.97	21.70 ± 1.95	29.66 ± 3.22	39.66 ± 2.35	42.03 ± 2.84	47.46 ± 2.68	*F*(2,24) = 191.000		<0.0001	*F*(1,24) = 30.8000		<0.0001	0.9994
	**TRN**	33.33 ± 3.07	34.79 ± 1.74	37.08 ± 3.09	50.42 ± 3.17	44.58 ± 4.25	53.96 ± 2.37	*F*(2,24) = 63.8900		<0.0001	*F*(1,24) = 52.4600		<0.0001	0.9944

		**1 month**		**3 months**		**6 months**		**PV conc.**		**Mito. volume**				
		**WT vs. KO**		**WT vs. KO**		**WT vs. KO**		**in WT neurons**		**increase 3 m**				
		***p*-value**		***p*-value**		***p*-value**								

	**DG**	0.9962		0.9999		0.9990		20 μM		11.3				
	**CA3**	0.9991		>0.9999		0.8843		20 μM		10.6				
	**CA1**	0.7731		0.9986		>0.9999		20 μM		13.4				
	**Striatum**	0.9446		0.0210*		0.7610		70 μM		109.2				
	**SSC**	0.9489		0.0496*		0.8271		90 μM		117.4				
	**mPFC**	0.9980		0.0163*		0.0042**		120 μM		130.8				
	**PC**	0.9736		0.0007***		0.1356		100 μM		58.2				
	**MLI**	0.9904		<0.0001****		0.0421*		570 μM		103.9				
	**TRN**	0.9721		<0.0001****		<0.0007***		750 μM		161.1				

**DHE**		**1 month**		**3 months**		**6 months**							
				
		**WT**	**KO**	**WT**	**KO**	**WT**	**KO**							**WT**

		**Mean ± SD**	**Mean ± SD**	**Mean ± SD**	**Mean ± SD**	**Mean ± SD**	**Mean ± SD**	**Effect of age**	**→**	***p*-value**	**Effect of genotype**	**→**	***p*-value**	***R*^2^ value**

	**DG**	5.73 ± 2.50	6.154 ± 1.82	6.068 ± 2.14	6.75 ± 1.33	6.67 ± 1.87	7.01 ± 1.92	*F*(2,24) = 0.5208		0.6006	*F*(1,24) = 0.4548		0.5065	0.9983
	**CA3**	6.80 ± 1.64	7.670 ± 1.43	8.155 ± 1.15	7.67 ± 2.33	8.93 ± 1.48	8.93 ± 2.44	*F*(2,24) = 2.2250		0.1299	*F*(1,24) = 0.0382		0.8466	0.9283
	**CA1**	5.86 ± 2.08	6.466 ± 1.52	6.552 ± 1.86	6.64 ± 2.18	7.33 ± 1.69	7.41 ± 2.00	*F*(2,24) = 1.0280		0.3729	*F*(1,24) = 0.1379		0.7137	0.9934
	**Striatum**	8.43 ± 1.22	9.29 ± 0.96	9.375 ± 1.51	12.23 ± 0.81	10.18 ± 0.71	12.23 ± 1.50	*F*(2,24) = 10.5300		0.0005	*F*(1,24) = 18.6200		0.0002	0.9745
	**SSC**	9.31 ± 1.45	9.71 ± 1.16	10.29 ± 1.05	12.57 ± 1.05	12.73 ± 1.01	14.04 ± 1.21	*F*(2,24) = 27.4600		<0.0001	*F*(1,24) = 9.7400		0.0046	0.9843
	**mPFC**	11.08 ± 0.89	11.69 ± 1.01	13.33 ± 0.77	15.06 ± 0.83	14.89 ± 0.51	15.93 ± 0.83	*F*(2,24) = 61.5200		<0.0001	*F*(1,24) = 13.7000		0.0011	0.9527
	**PC**	12.62 ± 1.11	12.67 ± 0.88	14.71 ± 0.97	18.14 ± 1.06	16.22 ± 1.03	17.91 ± 1.22	*F*(2,24) = 51.1200		<0.0001	*F*(1,24) = 20.0000		0.0002	0.9575
	**MLI**	18.81 ± 2.89	20.00 ± 1.75	19.49 ± 3.97	27.80 ± 2.57	22.54 ± 1.75	29.83 ± 3.51	*F*(2,24) = 14.2600		<0.0001	*F*(1,24) = 28.5300		<0.0001	0.9450
	**TRN**	17.08 ± 2.50	20.21 ± 2.28	22.08 ± 4.25	32.08 ± 3.63	24.17 ± 3.85	34.38 ± 4.71	*F*(2,24) = 23.6200		<0.0001	*F*(1,24) = 34.0400		<0.0001	0.8839

		**1 month**		**3 months**		**6 months**		**PV conc.**		**Mito. volume**				
		**WT vs. KO**		**WT vs. KO**		**WT vs. KO**		**in WT neurons**		**increase 3 m**				
		***p*-value**		***p*-value**		***p*-value**								

	**DG**	0.9993		0.9993		0.9998		20 μM		11.3				
	**CA3**	0.9713		0.9980		>0.9999		20 μM		10.6				
	**CA1**	0.9957		>0.9999		>0.9999		20 μM		13.4				
	**Striatum**	0.8955		0.0116*		0.1152		70 μM		109.2				
	**SSC**	0.9932		0.0503		0.5052		90 μM		117.4				
	**mPFC**	0.8550		0.0325*		0.3858		120 μM		130.8				
	**PC**	>0.9999		0.0004***		0.1566		100 μM		58.2				
	**MLI**	0.9853		0.0015**		0.0059**		570 μM		103.9				
	**TRN**	0.7530		0.0028**		0.0022**		750 μM		161.1				

Two-way ANOVA was performed for each brain region (upper parts) followed by Tukey’s multiple comparison (lower parts) for 8-oxo-dG and DHE signals. In addition, values of estimated PV concentrations in 3–5 months-old WT mice and mitochondria volume increase between 3-months old WT and KO mice are shown. The mean 8-oxo-dG and ox-DHE signal intensities were compared among groups using multivariate ANOVA followed by Tukey test for multiple comparisons. P-values > 0.05: not significant; *p < 0.05; **p < 0.01; ***p < 0.001; ****p < 0.0001. From WT 8-oxo-dG and ox-DHE data (1, 3, and 6 months), linear regression analysis was performed and *R*^2^ values are listed.

### Increased Oxidative Stress in PV−/− Pvalb Neurons Evident in Mice ≥ 3 Months

**FIGURE 2:**
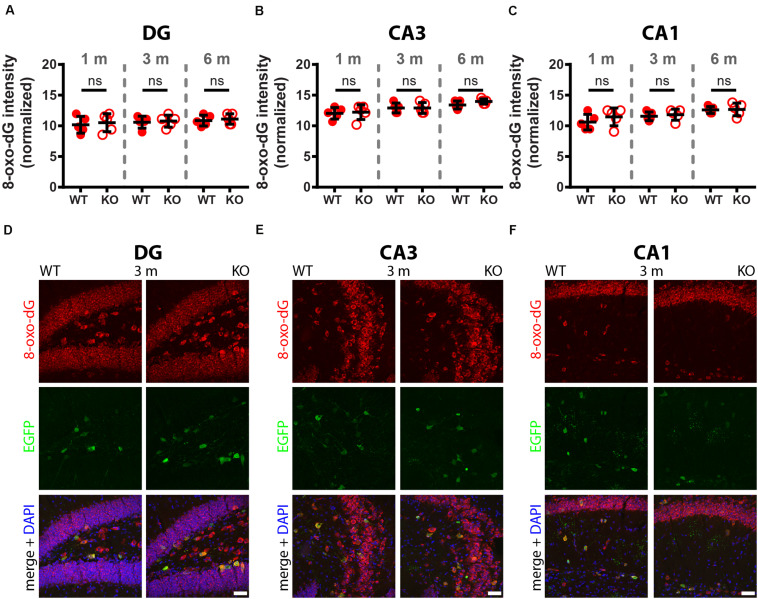
Comparison of 8-oxo-dG signal intensity between WT and KO mice in hippocampal regions of 1, 3, and 6-months old mice. The 8-oxo-dG fluorescence signal intensities (a.u.) analyzed in DG **(A)**, CA3 **(B)**, and CA1 **(C)** were normalized, taking into consideration the different sizes and morphologies of Pvalb neurons. Representative images with immunofluorescence labeling for 8-oxo-dG (red), EGFP (green) and merged images with DAPI (blue) are shown for DG **(D)**, CA3 **(E)** and CA1 **(F)** regions of 3-months old WT (left panels) and KO (right panels) mice. Scale bars: 50 μm. Each dot in the graphs represents the average obtained in 1 animal (5 mice per genotype) and at least 15 cells per animal resulting in > 75 cells per brain region (DG, CA3, CA1) and genotype. No significant differences were observed between WT and KO mice, ns – not significant. Values are reported in Table 1.

**FIGURE 3:**
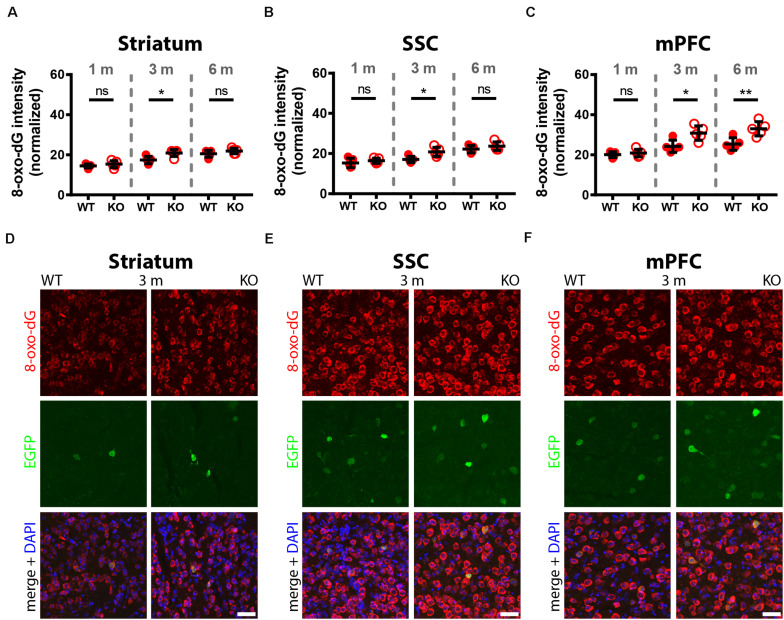
Comparison of 8-oxo-dG signal intensity in striatum, SSC and mPFC of 1, 3, and 6-months old WT and KO mice. Normalized 8-oxo-dG fluorescence signal intensities (a.u.) analyzed in striatum **(A)**, SSC **(B)** and mPFC **(C)** are shown. Representative images with immunofluorescence labeling for 8-oxo-dG (red), EGFP (green) and merged images with DAPI (blue) are shown in striatum **(D)**, SSC **(E),** and mPFC **(F)** of 3-months old WT (left panels) and KO (right panels) mice. Scale bars: 40 μm. Each dot in the graphs represents the average obtained in 1 animal (5 mice per genotype) and 10–15 cells per animal resulting in > 50 cells per brain region (striatum, SSC, mPFC) and genotype. ns not significant; *p < 0.05; **p < 0.01. Values are reported in Table 1.

**FIGURE 4:**
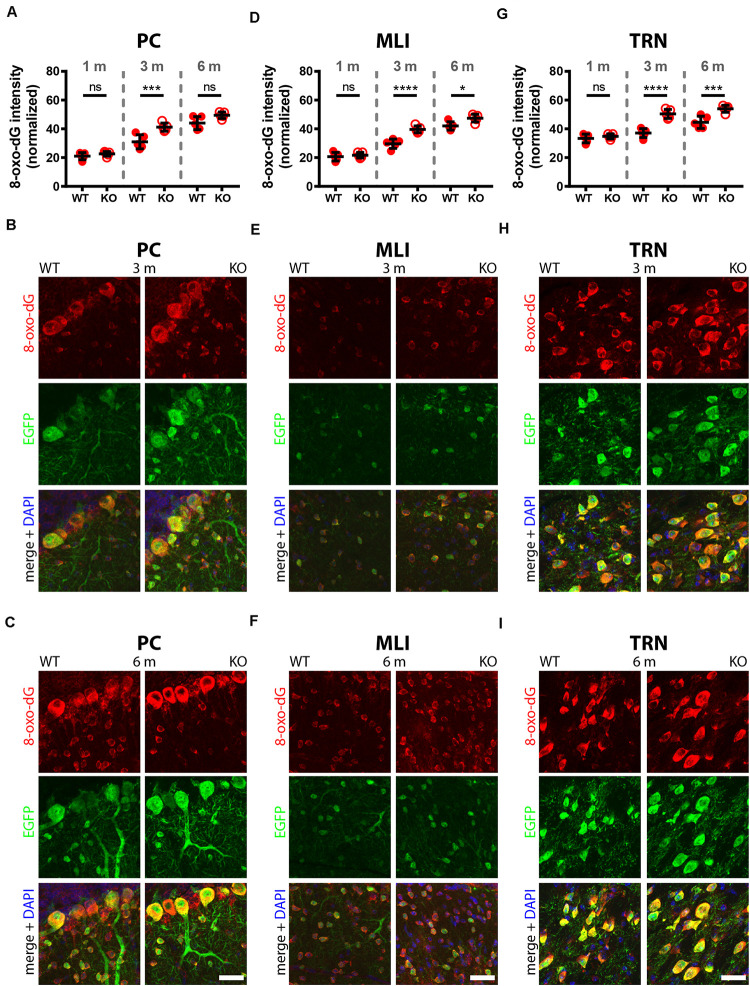
Comparison of 8-oxo-dG signal intensity in cerebellum (PC, MLI) and TRN of 1, 3, and 6-months old WT and KO mice. Normalized 8-oxo-dG fluorescence signal intensities (a.u.) analyzed in PC **(A)**, MLI **(D)**, and TRN Pvalb neurons **(G).** Representative images with immunofluorescence labeling for 8-oxo-dG (red), EGFP (green) and merged + DAPI (blue) in PC from 3 **(B)** and 6-months old **(C)** mice are shown. In **(E,F)** the same is shown for MLI; and in **(H,I)** for TRN Pvalb neurons in WT (left panels) and KO (right panels) mice. Scale bars: 30 μm. Each dot in the graphs represents the average obtained in 1 animal (5 mice per genotype) and at least 20 cells per animal resulting in > 100 cells per brain region (PC, MLI, TRN) and genotype. ns not significant; *p < 0.05; **p < 0.01; ***p < 0.001; ^****^p < 0.0001. Values are reported in Table 1.

A comparison of 8-oxo-dG signals between 1-month old WT and PV−/− mice (5 mice/genotype) revealed no significant differences in all nine investigated Pvalb neuron subpopulations as shown for hippocampus (Figure 2; DG, CA3, CA1), striatum, SSC and mPFC (Figure 3), cerebellum (PC, MLI) and TRN (Figure 4). In all cases signals were marginally (average: 5.2 ± 2.1% for all 9 Pvalb neuron populations), but not significantly increased in PV−/− Pvalb neurons. Measurement of acute oxidative stress accumulating during 18.5 h by the DHE method confirmed the results obtained by 8-oxo-dG staining. At 1 month, DHE signals were indistinguishable between WT and PV−/− Pvalb neurons in all investigated Pvalb neuron subpopulations (Supplementary Figure 3 and Table 1).

In 3-month old mice 8-oxo-dG signals were significantly higher in PV−/− mice in all Pvalb neurons with the exception of hippocampal (DG, CA3, CA1) Pvalb neurons, the latter shown in Figure 2. Quantitative immunofluorescence results and representative images for the 9 Pvalb neuron subpopulations in the analyzed brain regions are shown in Figures 2–4 and summarized in Table 1. Of note, the magnitude of the increase in 3-month old PV−/− mice was also in a graded manner and ranged from + 20% in striatal to + 36% in TRN Pvalb neurons (Table 1 and Supplementary Figure 4C). The magnitude in increase of oxidative stress strongly correlated with the PV concentration pertaining in the corresponding WT Pvalb neuron populations (compare to Supplementary Figure 3 in Janickova et al., 2020), and moreover with the increase in mitochondria volume in adult (3–5 months-old) PV-/- mice shown previously (Supplementary Figure 3 in Janickova et al., 2020). Thus, the higher the increase in mitochondria volume, the higher the oxidative stress in these PV-deficient Pvalb neurons. In addition to measuring (cumulative) oxidative stress by 8-oxo-dG staining, we used the DHE method to determine acute oxidative stress accumulated during a shorter time period (18.5 h). At 3 months, DHE signals were significantly increased in PV−/− Pvalb neurons in essentially all regions, except SSC (*p* = 0.0503) and hippocampus. In the latter low PV-expressing neurons, no differences existed between WT and PV−/− in all three subfields (DG, CA3, CA1; for all; *p* > 0.97), thus nearly perfectly replicating the results obtained by staining for 8-oxo-dG (Supplementary Figure 3 and Table 1).

In 6-months old mice, differences in 8-oxo-dG staining intensities persisted between WT and PV−/− Pvalb neurons, however effect sizes were generally smaller. Significant differences between genotypes were observed only in the groups of high PV-expressing Pvalb neurons including mPFC, MLI and TRN (Figures 3, 4 and Table 1). The smaller differences between genotypes in the other Pvalb neuron subpopulations (striatum, SSC, PC) were mostly resulting from a relatively larger (near-linear) increase in oxidative stress from 3- to 6-month old mice in the WT group, while the increase from 3 to 6 month in PV−/− Pvalb neurons leveled off. Thus, absence of PV leads to a different trajectory of ROS production, i.e., accelerated at 3 months and then approaching the WT condition at 6 months. The same tendency was also observed in mice subjected to DHE treatment. Using this approach, the further increase in DHE signal intensity in PV-deficient mice was significant in two Pvalb subpopulations (MLI and TRN), which are characterized by the highest PV expression levels, namely MLI (stellate and basket cells; [PV]: ∼570 μM (Eggermann and Jonas, 2012)) and TRN Pvalb neurons ([PV]: 600–900 μM, see Supplementary Figure 3 in Janickova et al. (2020).

### Trajectory of Increase in Mitochondria Density and Changes in Mitochondria Localization in TRN and Striatal Pvalb Neurons From 1 to 6 Months in WT and PV−/− Mice

**FIGURE 5:**
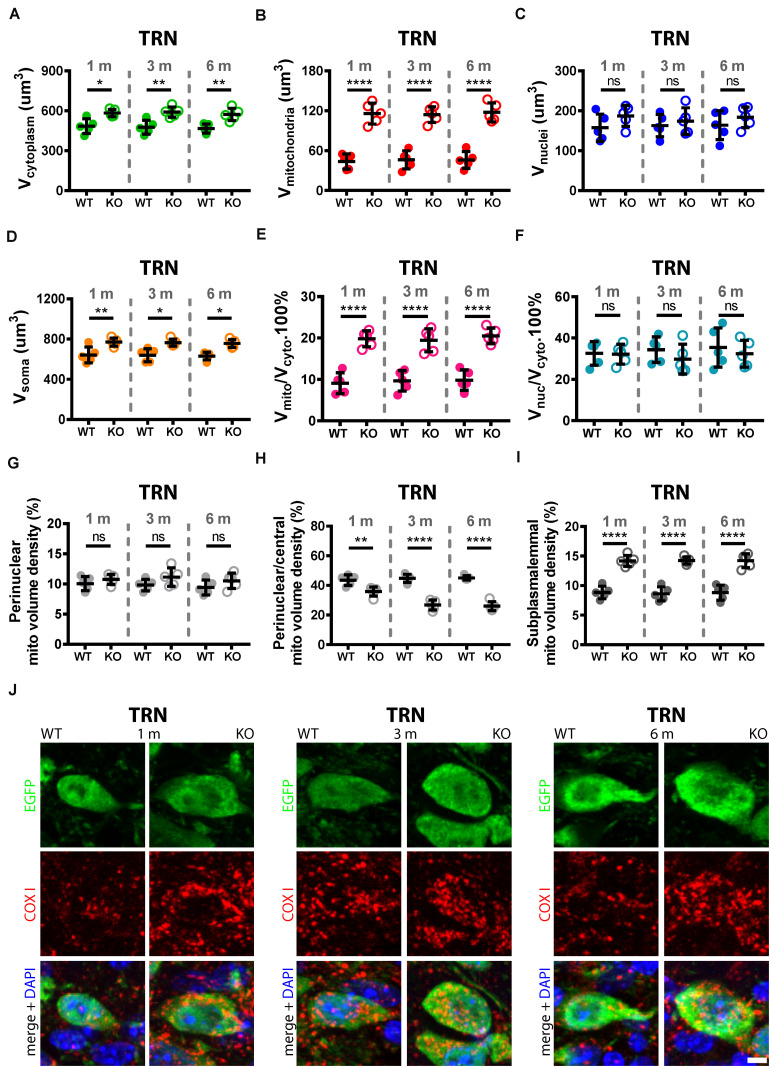
Quantitative morphological analyses of TRN Pvalb neurons. A comparison was carried out in 1, 3, and 6-months old WT and KO mice. Analyzed parameters included volume of cytoplasm **(A)**, volume of mitochondria **(B),** volume of nuclei **(C),** and volume of soma **(D)** in a given Pvalb neuron. Additional parameters were calculated, such as ratio V_*mitochondria*_/V_*cytoplasm*_ **(E)** and V_*nucleus*_/V_*cytoplasm*_ **(F)**. Quantitative analyses of the relative distribution of mitochondria within the cytoplasmic compartment in TRN Pvalb neurons **(G–I)**. Somata and nuclei of selected Pvalb neurons were identified based on EGFP and DAPI staining, respectively and the distribution of mitochondria from the center of the cell (border of nuclei) to the plasma membrane was partitioned into a set of 4 specified zones: 1 being the innermost (perinuclear) and 4 the outermost (subplasmalemmal). Quantitative analyses are shown for zone 1 (perinuclear; **G**), zone 2 (perinuclear/central **H**) and zone 4 (subplasmalemmal; **I**) mitochondrial volume density. Note the mitochondria shift from the perinuclear region to the periphery of the cells in KO mice. Representative confocal images **(J)** show EGFP-stained Pvalb neurons (green; cytoplasm), COX I (red; mitochondria) and DAPI (blue; nuclei) in 1, 3, and 6-months old WT (left panels) and KO (right panels) mice. Scale bar: 5 μm. Each dot in the graphs represents the average obtained in 1 animal (5 mice per genotype) and 20–30 cells per animal resulting in > 100 cells per TRN and genotype. ns: not significant; *p < 0.05; **p < 0.01; ***p < 0.001; ****p < 0.0001. Values are reported in Table 2.

**FIGURE 6:**
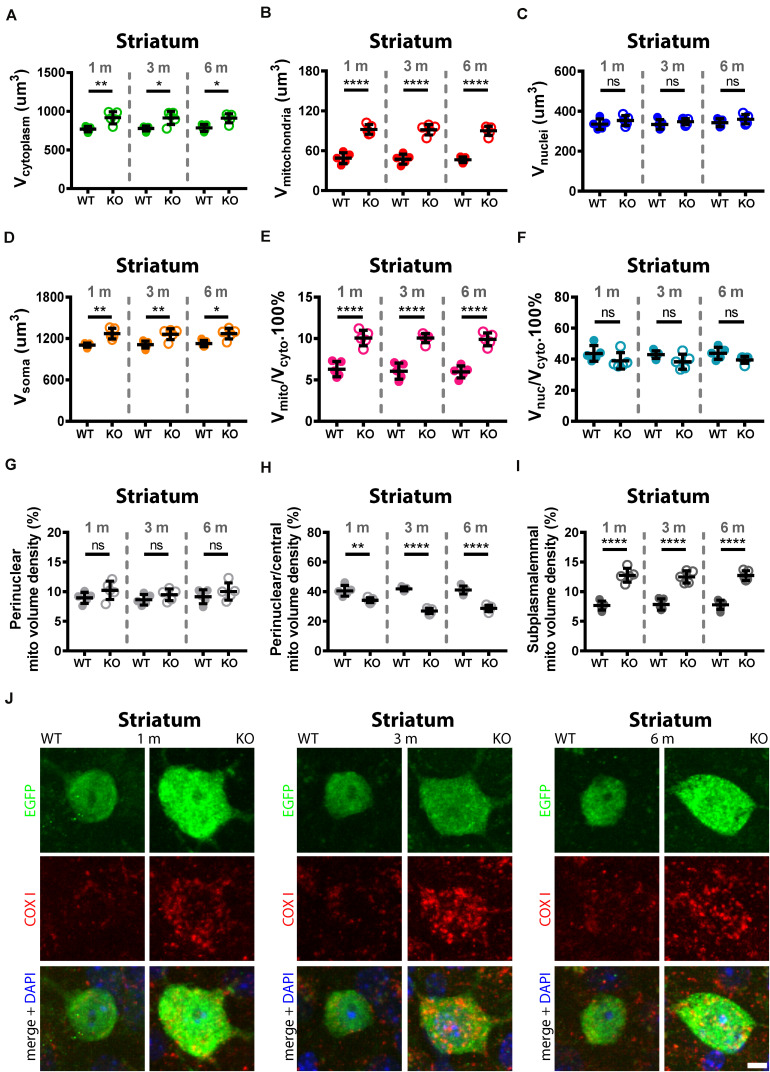
Quantitative morphological analyses of TRN Pvalb neurons. A comparison was carried out in 1, 3, and 6-months old WT and KO mice. Analyzed parameters included volume of cytoplasm **(A)**, volume of mitochondria **(B),** volume of nuclei **(C),** and volume of soma **(D)** in a given Pvalb neuron. Additional parameters were calculated, such as ratio V_*mitochondria*_/V_*cytoplasm*_ **(E)** and V_*nucleus*_/V_*cytoplasm*_ **(F)**. Quantitative analyses of the relative distribution of mitochondria within the cytoplasmic compartment in striatal Pvalb neurons **(G–I)**. Somata and nuclei of selected Pvalb neurons were identified based on EGFP and DAPI staining, respectively and the distribution of mitochondria from the center of the cell (border of nuclei) to the plasma membrane was partitioned into a set of 4 specified zones: 1 being the innermost (perinuclear) and 4 the outermost (subplasmalemmal). Quantitative analyses are shown for zone 1 (perinuclear; **G**), zone 2 (perinuclear/central **H**) and zone 4 (subplasmalemmal; **I**) mitochondrial volume density. Note the mitochondria shift from the perinuclear region to the periphery of the cells in KO mice. Representative confocal images **(J)** of EGFP-stained Pvalb neurons (green; cytoplasm), COX I (red; mitochondria) and DAPI (blue; nuclei) at all observed time points (1, 3, and 6 months) are shown for WT and KO mice. Scale bar: 5 μm. Each dot in the graphs represents the average obtained in 1 animal (5 mice per genotype) and 10–15 cells per animal resulting in > 50 cells per striatal region and genotype. ns: not significant; *p < 0.05; **p < 0.01; ***p < 0.001; ****p < 0.0001. Values are reported in Table 2.

While a significant increase in mitochondria density is present in adult (3–5-month old) PV−/− mice that strongly correlates with the PV concentration of various Pvalb neuron subpopulations (Janickova et al., 2020), nothing was previously known to when and to what extent the mitochondria increase occurred in younger mice. Moreover, whether changes took place also between 3 and 6 months was unknown. To close this knowledge gap, mitochondria volumes were determined in selected Pvalb neuron subpopulations at the age of 1, 3, and 6 months. For this analysis, we chose striatal and TRN Pvalb neurons in WT and PV−/− mice. The former, since we intended to correlate presumed mitochondria increases with increased branching observed already at PND18-24 (Wöhr et al., 2015) and persisting to adulthood (Janickova et al., 2020) and the latter, because the increase in mitochondria density and oxidative stress was largest in PV−/− TRN Pvalb neurons (Figure 4). In addition to determining soma mitochondria volume, we were interested in the distribution of mitochondria in the soma, since we had previously reported that in Purkinje cells of adult (3–5 months) PV−/− mice, the ∼40% increase in mitochondria volume was essentially restricted to a subplasmalemmal region of 1.5 μm (Chen et al., 2006). A comparison of the soma mitochondria density in TRN neurons revealed a significant increase in the order of 119% already at 1 month caused by the absence of PV. A similar increase persisted at 3 (+102%) and 6 (+109%) months and was essentially identical to the differences already prevailing at 1 month (Figure 5B). As observed previously (Janickova et al., 2020), absence of PV not only resulted in an increase in mitochondria volume, but also in the volume of the cytoplasm (Figure 5A) and subsequently of the entire soma (Figure 5D). Of note, the small increase in the volume of the nucleus was insignificant (Figure 5C). However even when considering the increase in the cytoplasm volume, the mitochondria density (V_*mitochondria*_/V_*cytoplasm*_) was still significantly higher (Figure 5E). The ratio V_*nucleus*_/V_*cytoplasm*_ (Figure 5F) was also not different indicating that the increase in the volume of the nucleus is approximately paralleled by the increase of the entire soma (Figure 5D). Qualitative similar results were observed in striatal Pvalb neurons (Figure 6), although effects were somewhat smaller, most probably related to the lower concentration of PV in striatal than in TRN Pvalb neurons (∼70 vs. ∼750 μM, respectively; Janickova et al., 2020). Nonetheless, the increase in mitochondrial density was substantial, in the order of + 60–65% and as in TRN Pvalb neurons already present in 1-month old mice deficient for PV. Thus, constitutive absence of PV during the entire neurodevelopment led to an increase in mitochondria density, which was already maximal at 1 month. Of note, the considerable increase in mitochondria volume (density) present at 1 month had no measurable effect on ROS production in the analyzed Pvalb neuron subpopulations in striatum and TRN. These findings are discussed below in detail with a focus on mechanisms implicated in the development of an ASD-like behavioral phenotype in PV−/− mice.

Importantly, the relative distribution of mitochondria within the cytoplasmic compartment was altered in the absence of PV in TRN (Figures 5G–I) and striatal (Figures 6G–I) Pvalb neurons. Clearly more mitochondria (+60%) accumulated in the subplasmalemmal region already at 1 month accompanied by relative mitochondria depletion mostly in the central cytoplasmic region that also persisted in mice of 3 and 6 months (values and statistics are listed in Table 2). Thus, PV-deficiency led to an overall increase in mitochondria and to an accumulation of subplasmalemmal mitochondria, both effects already maximal at 1 month indicating that absence of PV is rapidly sensed by Pvalb neurons and translated into an increase/redistribution of mitochondria.

**TABLE 2:** Quantitative results and statistical analyses of volumetric analysis and mitochondria position in four zones are shown as Mean ± SD in striatum and TRN.

TRN														

	Volumetric analysis													

			1 month		3 months		6 months							
					
			WT	KO	WT	KO	WT	KO						

			Mean ± SD	Mean ± SD	Mean ± SD	Mean ± SD	Mean ± SD	Mean ± SD	Effect of age	→	*p*-value	Effect of genotype	→	*p*-value
		**V_*cyto*_**	484.2 ± 55.3	582.8 ± 27.7	476.6 ± 52.4	590.2 ± 39.0	467.0 ± 34.4	572.4 ± 46.6	*F*(2,24) = 0.33040		0.7218	*F*(1,24) = 44.07		<0.0001
		**V_*mito*_**	43.6 ± 11.3	115.8 ± 15.8	46.2 ± 13.5	114.2 ± 11.7	46.0 ± 12.8	117.6 ± 14.3	*F*(2,24) = 0.06775		0.9347	*F*(1,24) = 210.50		<0.0001
		**V_*nuclei*_**	157.6 ± 34.0	187.2 ± 26.4	162.8 ± 28.2	174.2 ± 33.3	163.8 ± 36.2	183.6 ± 25.9	*F*(2,24) = 0.07659		0.0926	*F*(1,24) = 3.22		0.0853
		**V_*soma*_**	641.8 ± 79.6	770.0 ± 39.4	639.4 ± 65.0	764.4 ± 34.6	630.8 ± 38.7	756.0 ± 39.0	*F*(2,24) = 0.14990		0.8616	*F*(1,24) = 43.89		<0.0001
		**V_*mito*_/V_*cyto*_**	9.1 ± 2.5	19.8 ± 1.9	9.7 ± 2.5	19.4 ± 2.8	9.8 ± 2.5	20.5 ± 1.9	*F*(2,24) = 0.26990		0.7658	*F*(1,24) = 144.10		<0.0001
		**V_*nuc*_/V_*cyto*_**	32.6 ± 5.7	32.2 ± 4.8	34.4 ± 6.2	29.79 ± 7.2	35.4 ± 9.5	32.4 ± 6.5	*F*(2,24) = 0.21200		0.8105	*F*(1,24) = 1.13		0.2972

			**1 month**		**3 months**		**6 months**							
			**WT vs. KO**		**WT vs. KO**		**WT vs. KO**							
			***p*-value**		***p*-value**		***p*-value**							

		**V_*cyto*_**	0.0171*		0.0047**		0.0096**							
		**V_*mito*_**	<0.0001****		<0.0001****		<0.0001****							
		**V_*nuclei*_**	0.6593 ns		0.9913 ns		0.9092 ns							
		**V_*soma*_**	0.0081**		0.0102*		0.0101*							
		**V_*mito*_/V_*cyto*_**	<0.0001****		<0.0001****		<0.0001****							
		**V_*nuc*_/V_*cyto*_**	>0.9999 ns		0.8934 ns		0.9803 ns							

	**Mito volume density**													

			**1 month**		**3 months**		**6 months**							
					
			**WT**	**KO**	**WT**	**KO**	**WT**	**KO**						

			**Mean ± SD**	**Mean ± SD**	**Mean ± SD**	**Mean ± SD**	**Mean ± SD**	**Mean ± SD**	**Effect of age**	**→**	***p*-value**	**Effect of genotype**	**→**	***p*-value**

		Zone_*I*_	10.1 ± 1.2	10.8 ± 0.8	9.8 ± 1.0	11.1 ± 1.5	9.4 ± 1.2	10.5 ± 1.3	*F*(2,24) = 0.54270		0.5881	*F*(1,24) = 5.76		0.0245
		Zone_*II*_	43.5 ± 3.5	35.9 ± 3.1	44.8 ± 2.8	26.8 ± 3.3	45.1 ± 1.5	26.0 ± 3.0	*F*(2,24) = 6.27000		0.0064	*F*(1,24) = 191.50		<0.0001
		Zone_*III*_	37.5 ± 3.2	39.2 ± 3.5	36.8 ± 2.6	47.9 ± 3.2	36.7 ± 2.1	49.2 ± 4.0	*F*(2,24) = 6.09300		0.0072	*F*(1,24) = 52.09		<0.0001
		Zone_*IV*_	8.8 ± 1.1	14.2 ± 0.9	8.6 ± 1.2	14.2 ± 0.6	8.8 ± 1.3	14.2 ± 1.2	*F*(2,24) = 0.02404		0.9763	*F*(1,24) = 199.50		<0.0001

			**1 month**		**3 months**		**6 months**							
			**WT vs. KO**		**WT vs. KO**		**WT vs. KO**							
			***p*-value**		***p*-value**		***p*-value**							

		Zone_*I*_	0.9340 ns		0.5091 ns		0.6882 ns							
		Zone_*II*_	0.0048**		<0.0001****		<0.0001****							
		Zone_*III*_	0.9639 ns		0.0002***		<0.0001****							
		Zone_*IV*_	<0.0001****		<0.0001****		<0.0001****							

**Volumetric analysis**	

			**1 month**		**3 months**		**6 months**							
					
			**WT**	**KO**	**WT**	**KO**	**WT**	**KO**						

			**Mean ± SD**	**Mean ± SD**	**Mean ± SD**	**Mean ± SD**	**Mean ± SD**	**Mean ± SD**	**Effect of age**	**→**	***p*-value**	**Effect of genotype**	**→**	***p*-value**

		**V_*cyto*_**	769.0 ± 34.9	916.0 ± 78.5	777.6 ± 32.6	914.4 ± 85.4	784.8 ± 44.8	910.2 ± 59.4	*F*(2,24) = 0.01856		0.9816	*F*(1,24) = 39.33		<0.0001
		**V_*mito*_**	48.8 ± 8.2	92.0 ± 7.3	47.2 ± 7.6	91.6 ± 7.9	46.4 ± 4.2	90.0 ± 6.9	*F*(2,24) = 0.23790		0.7901	*F*(1,24) = 281.3		<0.0001
		**V_*nuclei*_**	335.0 ± 26.2	353.8 ± 24.3	333.8 ± 24.0	347.2 ± 15.1	342.6 ± 19.7	359.8 ± 23.7	*F*(2,24) = 0.58060		0.5672	*F*(1,24) = 4.027		0.0562
		**V_*soma*_**	1104 ± 27.6	1270 ± 76.0	1111 ± 50.6	1262 ± 78.9	1127 ± 45.3	1270 ± 76.5	*F*(2,24) = 0.12400		0.8839	*F*(1,24) = 45.24		<0.0001
		**V_*mito*_/V_*cyto*_**	6.3 ± 0.9	10.1 ± 0.9	6.1 ± 1.0	10.04 ± 0.6	6.0 ± 0.7	9.9 ± 0.8	*F*(2,24) = 0.22840		0.7975	*F*(1,24) = 166.1		<0.0001
		**V_*nuc*_/V_*cyto*_**	43.7 ± 5.1	38.9 ± 5.2	42.9 ± 2.4	38.3 ± 4.8	43.8 ± 3.9	39.6 ± 2.2	*F*(2,24) = 0.16530		0.8486	*F*(1,24) = 8.981		0.0063

			**1 month**		**3 months**		**6 months**							
			**WT vs. KO**		**WT vs. KO**		**WT vs. KO**							
			***p*-value**		***p*-value**		***p*-value**							

		**V_*cyto*_**	0.0078**		0.0148*		0.0297*							
		**V_*mito*_**	<0.0001****		<0.0001****		<0.0001****							
		**V_*nuclei*_**	0.7699 ns		0.9311 ns		0.8277 ns							
		**V_*soma*_**	0.0037**		0.0096**		0.0152*							
		**V_*mito*_/V_*cyto*_**	<0.0001****		<0.0001****		<0.0001****							
		**V_*nuc*_/V_*cyto*_**	0.4699 ns		0.5063 ns		0.6045 ns							

	**Mito volume density**													

			**1 month**		**3 months**		**6 months**							
					
			**WT**	**KO**	**WT**	**KO**	**WT**	**KO**						

			**Mean ± SD**	**Mean ± SD**	**Mean ± SD**	**Mean ± SD**	**Mean ± SD**	**Mean ± SD**	**Effect of age**	**→**	***p*-value**	**Effect of genotype**	**→**	***p*-value**

		Zone_*I*_	9.0 ± 0.9	10.2 ± 1.6	8.6 ± 0.9	9.5 ± 1.0	9.1 ± 1.2	10.0 ± 1.5	*F*(2,24) = 0.64150		0.5353	*F*(1,24) = 5.166		0.0323
		Zone_*II*_	40.6 ± 3.6	34.2 ± 1.6	41.9 ± 1.3	26.9 ± 1.8	41.1 ± 2.7	28.7 ± 2.1	*F*(2,24) = 4.66200		0.0195	*F*(1,24) = 177.3		<0.0001
		Zone_*III*_	42.8 ± 4.6	42.8 ± 2.6	41.6 ± 2.2	51.2 ± 2.9	41.9 ± 2.8	48.5 ± 3.3	*F*(2,24) = 3.27200		0.0554	*F*(1,24) = 21.73		<0.0001
		Zone_*IV*_	7.7 ± 0.8	12.8 ± 1.2	7.8 ± 0.9	12.5 ± 1.0	7.8 ± 0.8	12.7 ± 0.8	*F*(2,24) = 0.02351		0.9768	*F*(1,24) = 207.6		<0.0001

			**1 month**		**3 months**		**6 months**							
			**WT vs. KO**		**WT vs. KO**		**WT vs. KO**							
			***p-*value**		***p*-value**		***p*-value**							

		Zone_*I*_	0.5666 ns		0.8726 ns		0.8498 ns							
		Zone_*II*_	0.0027**		<0.0001****		<0.0001****							
		Zone_*III*_	>0.9999 ns		0.0009***		0.0321*							
		Zone_*IV*_	<0.0001****		<0.0001****		<0.0001****							

Two-way ANOVA was performed for each parameter followed by Tukey’s multiple comparison. The values were compared among groups using multivariate ANOVA followed by Tukey test for multiple comparisons. ns, not significant; *p < 0.05; **p < 0.01; ***p < 0.001, ****p < 0.0001.

## Materials and Methods

### Animals

Two transgenic lines were used in this study: B6.Tg(Pvalb-EGFP)^1*Hmon*^ mice (WT) expressing normal levels of PV and the enhanced green fluorescent protein (EGFP) selectively in Pvalb neurons (Meyer et al., 2002); the second line B6.Pvalb^*tm1*1*Swal*^ x B6Tg(Pvalb-EGFP)^1*Hmon*^ (KO) is additionally devoid of functional *Pvalb* alleles (Orduz et al., 2013). Both lines have been used in previous studies aimed at elucidating the role of PV in Pvalb neurons (Orduz et al., 2013; Janickova et al., 2020). All together 30 female mice were used in this study, sacrificed when they reached 1, 3, and 6 months of age; five mice per age group and genotype. Mice were group-housed in the rodent facility at the University of Fribourg, Switzerland in temperature-controlled rooms (24°C), with 12:12 h light/dark cycle interval. All animals had free access to water and were fed *ad libitum*. Experiments were performed according to institutional guidelines of the present Swiss law and the European Communities Council Directive of 24 November 1986 (86/609/EEC). The authorization number for housing of mice is H-04.2012-Fr and for the experiments 2016_37_FR. All experiments were approved by the Cantonal Veterinary Office (Canton of Fribourg, Switzerland).

### *In vivo* Injections of the Fluorescent Dye Dihydroethidium

To analyze superoxide production in specific brain regions, we used the fluorescent probe dihydroethidium (DHE) (Thermo Fisher Scientific, Switzerland, Cat # D11347). The characterization and validation of DHE for whole animal fluorescence imaging has been successfully carried out before (Hall et al., 2012). When administered systemically, DHE distributes rapidly into the various tissues including the brain (Murakami et al., 1998; Quick and Dugan, 2001). There the uncharged lipophilic compound DHE is converted by superoxide radicals to the positively charged product ox-DHE (Barbacanne et al., 2000; Fink et al., 2004) and, if not oxidized, is cleared from tissues and excreted in the urine (Hall et al., 2012). Importantly, ox-DHE is retained in the brain for a sustained period of time due to its charge (Quick and Dugan, 2001). This then allows for the quantification of the amount of superoxide produced over a defined period of time in a given tissue by measurement of ox-DHE (Hall et al., 2012). Mice at the ages of 1, 3, and 6 months received two serial intraperitoneal injections of freshly prepared DHE solution (27 mg/kg) at 30 min intervals (the first bolus was generally injected at ∼16:00 and the second injection at 16:30). Eighteen hours after the second DHE injection (∼10:30), animals were anesthetized with a lethal dose of Esconarkon^®^ (300 mg/kg body weight; Streuli Pharma AG, Uznach, Switzerland) and perfused as described below.

### Tissue Preparation and Immunohistochemistry

Mice were anesthetized with 300 mg/kg body weight Esconarkon^®^ (Streuli Pharma AG, Uznach, Switzerland) and perfused using 0.9% NaCl, followed by perfusion with 4% PFA in 0.9% NaCl. Brains were removed, post-fixed for 24 h in 4% PFA in TBS and cryopreserved in 30% sucrose-TBS (0.1M, pH 7.3) at 4°C, as described before (Lauber et al., 2016). From the entire brains of each mouse coronal sections (40 μm) were cut in the rostro-caudal direction using a freezing microtome (Leica SM2010R, Switzerland) as described before (Filice et al., 2016). For the 9 regions of interest embracing the somatosensory cortex (SSC) and mPFC, striatum, TRN, hippocampal regions DG, CA3, CA1 and cerebellar Purkinje cell layer (Purkinje cells) and molecular layer (interneurons; MLI), three sections per mouse and brain region of interest were selected according to the Allen brain atlas and Paxinos Franklin atlas, following the rules of stereological systematic random sampling principles (for details, see, Filice et al., 2016). Selected brain slices were used immediately for immunohistochemistry, unused brain sections were stored in antifreeze solution at −20°C. Free-floating brain sections containing all the regions of interest were first blocked for 1 h at RT in TBS (0.1M, pH 7.3) containing 10% bovine serum albumin (BSA) and 0.4% Triton X-100. Afterward, slices were rinsed three times in TBS. The following primary antibodies were used: rabbit anti-EGFP (Molecular Probes, Thermo Fisher Scientific, Switzerland, Cat # A6455; dilution 1:1000), anti-8-oxo-dG (mouse monoclonal; AMS Biotechnology, Biogio-Lugano, Switzerland, Clone 2E2, Cat # 4354-MC-050, dilution 1:350) as reported before (Cabungcal et al., 2019). 8-oxo-7,8-dihydro-20-deoxyguanine (8-oxo-dG) is a product of DNA oxidation, serving as marker for the evaluation of oxidative stress. Incubation with primary antibodies was performed for 48 h at 4°C (Cabungcal et al., 2019). On parallel sections, COX I antibody (mouse monoclonal anti-cytochrome oxidase 1), clone COX 111 (Molecular Probes, Invitrogen AG, Switzerland, Cat # 35-810; dilution 1:500) and rabbit anti-EGFP antibody (Molecular Probes, Thermo Fisher Scientific, Switzerland, Cat # A6455; dilution 1:1000) were used overnight at 4°C. Sections were rinsed twice with TBS and once with Tris-HCl 0.1M, pH 8.2 for 5 min each and then incubated with anti-rabbit Alexa488-conjugated antibody (Life Technologies, Thermo Fisher Scientific, Switzerland; 1:450 dilution), anti-mouse Cy3-conjugated antibody (Jackson Immunoresearch, Suffolk, United Kingdom) and anti-mouse Alexa647-conjugated antibody (Life Technologies, Thermo Fisher Scientific, Switzerland, 1:450 dilution) used as secondary antibodies for 4 h at 4°C. All sections were washed three times with TBS and nuclei were stained with DAPI (LuBio Science GmbH, Switzerland; 1:1000 dilution), during the last 5 min of the incubation period with secondary antibody in TBS. After final washing, slides were transferred onto MENZEL-GLÄSER SUPERFROST^®^ (Thermo Fisher Scientific, Switzerland) and coverslipped with Hydromount (National Diagnostics, Atlanta, GA, United States).

### Whole Slide Scanning

Brain sections were scanned by a fully automated slide scanner NanoZoomer 2.0-HT (Hamamatsu Photonics K.K, Switzerland). Fluorescence images were acquired using the mercury lamp unit L11600-05 and for DAPI, FITC, and Cy3 fluorescence imaging, a filter cube with excitation filters (λ_*Ex*_: 387, 485, and 560 nm) and emission filters (λ_*Em*_ 410, 504, 582 nm) was used. All sections were scanned along the z-axis with a 1.4 μm interval with a 20X objective (numerical aperture 0.75) and using 0.46 μm/pixel as scale factor. Images were exported using NDP.view2 Image viewing software (NanoZoomer, Hamamatsu Photonics K.K, U12388-01) and adjusted in Fiji software (RRID:SCR_002285), an open-source platform for image analysis of biological samples.

### Confocal Microscopy and Image Post-processing

Mounted brain sections were examined by laser scanning confocal microscopy using a Leica TCS-SP5 instrument (Leica Microsystems, Inc., Buffalo Grove, IL, United States) equipped with a 40X oil-immersion APO plan objective (numerical aperture 1.3). Fluorescence of oxidized DHE was obtained by excitation at λ_*Ex*_ 516 nm and recording the emission at λ_*Em*_ 570–600 nm. For EGFP-stained neurons, confocal settings were λ_*Ex*_ 477 nm, λ_*Em*_ 485–510. Specific brain regions were scanned along the z-axis with a 1.4 μm interval as described before (Behrens et al., 2008) with constant acquisition parameters; 1240 × 1240 pixels, 200 Hz scan speed and 1AU pinhole diameter. Fluorescence of 8-oxo-dG was acquired by excitation at λ_*Ex*_ 561 nm and recording the emission at λ_*Em*_ 565–660 nm. For volumetric analyses confocal settings were as follows: λ_*Ex*_ 405 nm, λ_*Em*_ 410–480 (DAPI), EGFP λ_*Ex*_ 488 nm, λ_*Em*_ 495–600 nm (EGFP) and λ_*Ex*_ 633 nm, λ_*Em*_ 640–800 (COX I). Sections were scanned along the z-axis at 0.42-μm step intervals, as described previously (Janickova et al., 2020), with constant acquisition parameters; 1240 × 1240 pixels, 200 Hz scan speed and 1AU pinhole diameter. After acquisition, all images were deconvoluted using the Huygens deconvolution software (Scientific Volume Imaging, Netherlands) to eliminate blurring and noise and filtered with a Gaussian filter to remove unwanted background noise and to sharpen cell profile contours.

### Image Analysis

Coronal brain sections from 30 mice, 15 WT and 15 KO (*n* = 5 animals per genotype and age) were used. From each brain, nine specific regions (DG, CA3, CA1, striatum, mPFC, SSC, PC, MLI, and TRN) were selected. From sections containing the above brain regions, three parallel sections were collected following stereological systematic random sampling principles (West et al., 1991). Each section (40 μm) was scanned by laser scanning confocal microscope and z-stacks were acquired. From each defined brain region, a single field was randomly selected and only neuron somata completely positioned within the xyz-stack volume were analyzed, i.e., 3–10 neurons per section. The number of analyzed neurons per section fulfilling the criteria varied based on the density of Pvalb neurons in each brain region; e.g., a relatively low density of Pvalb neurons is characteristic for striatum and a high density of Pvalb neurons is typical for cerebellum and TRN (for more details, see Supplementary Figure 1 in Janickova et al., 2020). The minimum number of neurons that were analyzed from the three sections of each brain region is listed in the figure legends of Figures 2–4. To quantify the overall 8-oxo-dG or DHE fluorescence signal intensity within the ROI, we first used Imaris 9.5.1 software (Bitplane, AG, Switzerland, RRID:SCR_007370) to confirm that the soma of an EGFP^+^ neuron is completely within the boundaries of the xyz volume. Next the proportion of all 8-oxo-dG or DHE immunolabeled voxels contained in the center 8 images of the z-stacks and the mean fluorescence intensity was calculated as previously reported (Cabungcal et al., 2019) using the LAS AF software (Leica Application Suite X, RRID:SCR_013673). We used maximum intensity projections to obtain the values for ROIs, a ROI was drawn using the “free hand tool” based on the EGFP^+^ neuron morphology. For background subtraction, a similar-sized ROI within the tissue characterized by weak and diffuse staining not overlapping with EGFP or clearly discernible 8-oxo-dG or DHE fluorescence signals was randomly selected from each microscopy field. The values obtained after background subtraction from all analyzed neurons from each section were averaged resulting in a single value per animal and brain region. Since fluorescence intensity values were obtained from maximal density projections (z-stacks) and the morphology of the different Pvalb neuron subpopulations is quite variable, a correction factor based on Pvalb neuron morphology was calculated (Janickova et al., 2020). Briefly, assuming that neuron somata and nuclei can be approximated by spheres, from the volume of the entire soma and the nucleus, the radii of these two spheres (r2: soma; r1: nucleus) were calculated (see Supplementary Table S1 in Janickova et al., 2020). The difference in the radii is then the thickness (d) of the shell comprising the cytoplasmic volume. Hence, fluorescence values listed in Table 1 are the ones after applying the d-factor correction. To determine the volume of soma, nucleus and mitochondria of EGFP^+^ neurons entirely localized within z-stack images, the ‘Cell’ and ‘Surface’ module of the Imaris software was used as described before (Janickova et al., 2020). The volume of the cytoplasm, nuclei and mitochondria were calculated for each neuron using the same parameter and algorithm settings. Statistical analyses were performed using the ‘Imaris Measurement Pro’ module as described previously (Lichvarova et al., 2019).

### Mitochondria Distribution in Pvalb Neuron Somata

To analyze mitochondria distribution in the somata of Pvalb neurons, the CellProfiler^TM^ (Cell Image Analysis software, Broad Institute Imaging Platform, RRID:SCR_007358) with the ‘Measure Object Intensity Distribution’ module was used. This module allows to measure the spatial distribution of intensities within each identified object. First, morphologically fully intact Pvalb neuron somata contained in the z-stack were selected and somata and nuclei of selected neurons were identified based on EGFP and DAPI staining, respectively. Next, the distribution from the center of the cell (nucleus) to the edge (i.e., the plasma membrane of a selected neuron soma) was partitioned into a set of four specified zones excluding the nucleus: 1 being the innermost and 4 the outermost for details, see CellProfiler. The software then measured automatically the fraction of total staining intensity in an object at a given radius. Data are shown as perinuclear mitochondrial volume (zone1), perinuclear/central mitochondrial volume (zone 2) and subplasmalemmal mitochondrial volume (zone 4).

### Statistical Analysis

GraphPad Prism 7.05 software (RRID:SCR_002798) was used for statistical analysis. Two-way multivariate ANOVA followed by Tukey multiple comparison test was performed in order to compare the ratios between PV-EGFP (WT) and PVKO-EGFP (PV−/− or KO) mice and age in different brain regions. For all experiments a *p*-value < 0.05 was considered as statistically significant. Values are expressed as mean ± SD. The mean 8-oxo-dG and ox-DHE signal intensities were compared among groups using multivariate ANOVA followed by Tukey test for multiple comparisons. Detailed statistical analysis with mean ± SD, age and genotype as factors, as well as exact *p-*values are reported in Tables 1, 2.

## Discussion

Release of mitochondrial ROS (mROS) is suggested to have evolved as a communication system linking mitochondrial function with physiological cellular processes to maintain homeostasis in the brain and to sustain adaptation to stress (reviewed in Sena and Chandel, 2012). In the brain mROS act as physiological modulators of signaling pathways and transcription factors involved in cell proliferation, differentiation and maturation (e.g., in axon formation); in mature neurons mROS also participate in the regulation of synaptic plasticity [details are provided in Figure 1 and the references cited in the review by Beckhauser et al. (2016)]. Converse to their important physiological functions, elevated ROS levels causing an imbalance between ROS production and antioxidant defenses (principally provided by glutathione), as found in post-mortem brain tissue of children with ASD (Siddiqui et al., 2016), and adult schizophrenia patients (Tosic et al., 2006) might be implicated in ASD and schizophrenia etiology. The link between redox dysregulation and schizophrenia has been investigated in a mouse model, i.e., in Gclm−/− mice deficient for the glutamate cysteine ligase modifier subunit, the rate-limiting enzyme for glutathione biosynthesis (Cabungcal et al., 2019). The early-onset developmental redox dysregulation caused by constitutive absence of Gclm most strongly affects the Pvalb neuron circuitry evidenced by increased 8-oxo-dG staining in VVA^+^ Pvalb neurons. The authors provide evidence “that PV neurons located in different cortical and sub-cortical brain regions exhibit selective vulnerability to oxidative stress during different phases of neurodevelopment” (see Figure 6 in Cabungcal et al., 2019). Potential explanations for these region-specific variances include “differences in (1) maturation time course of PV neurons and their PNN, (2) neuronal and metabolic activity, (3) antioxidant capacity of PV neurons and their neighbored cells, (4) levels of catecholamines (dopamine, noradrenaline)” and possibly temporal changes in functional connectivity between brain regions (Cabungcal et al., 2019).

Results from our longitudinal study indicate that the magnitude and moreover, the age-dependent increase in oxidative stress in Pvalb neurons is highly correlated with the PV concentration in those neurons of WT mice (Supplementary Figure 4). In absence of PV, the elevated oxidative stress is strongly correlated with the PV deficiency-induced increase in mitochondria volume (density) reported before (Janickova et al., 2020) and shown here for TRN and striatal Pvalb neurons (Figures 5, 6). Compared to WT mice enhanced oxidative stress was detectable in PV−/− mice from 3 months on, although the increase in mitochondrial volume was already maximal at 1 month evidenced in striatal and TRN Pvalb neurons. Yet, at this age oxidative stress was not increased in absence of PV. In summary, the larger the increase in mitochondria volume caused by the absence of PV in Pvalb neurons, the larger the oxidative stress, evident only in older (>3 months) mice.

Inverse (antagonistic) regulation of PV and mitochondria is observed in several *in vitro* and *in vivo* models (reviewed in Schwaller, 2020) including Pvalb neurons of PV−/− mice (Janickova et al., 2020). In this study, a strong correlation between the PV deficiency-induced increase in mitochondria and the prevailing PV concentration in Pvalb neurons of WT mice was observed. The increase in mitochondria is likely regulated by the master regulator of mitochondria biosynthesis PGC-1α, as previously shown in fast-twitch muscle of PV−/− mice, where mitochondria volume is also upregulated in absence of PV (Ducreux et al., 2012). Of note, PGC-1α overexpression also increases expression of ROS defense systems likely to maintain redox balance (St-Pierre et al., 2006). In line, in MDCK (epithelial) cells mRNA levels of *Ucp2* are higher in PV-negative control cells (a proxy measure for PV-deficient Pvalb cells) compared to PV-overexpressing cells characterized by a decreased mitochondria volume (Henzi and Schwaller, 2015). The same holds true for CG4 cells, where *Ucp2* levels are higher in PV-negative control cells than in PV-expressing CG4 cells (L. Janickova, unpublished). Upregulation of uncoupling proteins decreases the mitochondrial membrane potential ΔΨ and concomitantly is expected to reduce mROS production. Thus, possibly elevated ROS defense systems [as found in the MDCK cell model (Henzi and Schwaller, 2015) and CG4 cells] despite a significant increase in mitochondria volume in PV−/− Pvalb neurons of 1-month old mice might prevent elevated ROS production at this age.

Thus, we propose the following timeline for events taking place in Pvalb neurons, if PV expression is constitutively lacking in PV−/− mice. Absence of PV is rapidly detected by Pvalb neurons possibly sensed as changes in the shape of Ca^2+^ transients shown to occur in Purkinje cells (Schmidt et al., 2003), MLI (Collin et al., 2005) and in a large Pvalb neuron presynapse, the calyx of Held (Muller et al., 2007). At the functional level this results in increased paired-pulse facilitation (PPF) as seen at synapses involving cerebellar (Caillard et al., 2000; Collin et al., 2005), striatal (Orduz et al., 2013), and hippocampal (Vreugdenhil et al., 2003) Pvalb neurons. These alterations are then leading to modifications in excitation-transcription coupling inducing several changes including mitochondria biosynthesis resulting in mitochondria that are tuned to optimally contribute to slow Ca^2+^ buffering. This mitochondria-mediated process is energy-expensive and can only be maintained by increased ATP production (Palmieri and Persico, 2010) paralleled by increases in mROS production (for reviews, see, Devine and Kittler, 2018; Giorgi et al., 2018) that are manifest in PV−/− mice only at older (>3 months) age. We assume that although increased mitochondria volume (density) in absence of PV is already maximal at 1 month, the possibly concomitant increase in ROS defense systems including UCP2 might be sufficient to prevent augmentation of oxidative stress at this age.

Of relevance, transcriptional regulation of PV is mediated not by the prototypical CaMKII or IV, but by the “atypical” γCaMKI (Cohen et al., 2016). In line, *CAMK1G* mRNA is among the most strongly downregulated transcripts in cortical samples from ASD individuals initially reported in Parikshak et al. (2016) and further analyzed in Schwede et al. (2018), thus providing the link to decreased levels of *PVALB* mRNA and elevated mitochondrial genes in human ASD including *UCP2*. The elevated mitochondria density previously determined in Pvalb neurons of 3–5 months-old mice is ranging from ∼5% (hippocampus) to ∼108% (TRN) and is approximately proportional to the PV concentration prevailing in WT Pvalb neurons (see Supplementary Figure 3 in Janickova et al., 2020). Of importance, in 1-month old PV−/− mice, when PV expression has reached adult levels (as shown in cerebellar Pvalb neurons; see Figure 2 in Collin et al., 2005), the increase in mitochondria density is already maximal: ∼60–65% in striatal and ∼115% in TRN Pvalb neurons. Of importance, in none of the investigated Pvalb neuron subpopulations, an increase in oxidative stress is evident at this time point. However, at the behavioral level PV−/− mice show ASD-like core symptoms at PND25–30 (Wöhr et al., 2015). This essentially precludes oxidative stress as causative for the ASD-like behavioral phenotype of PV−/− mice. It rather supports the hypothesis that altered Ca^2+^ signals in PV−/− Pvalb neurons subtly modify synaptic plasticity likely translating into changes in neuron ensemble synchrony and oscillations. Notwithstanding additional alterations affecting Pvalb neuron firing caused by the absence of PV might be implicated as well: e.g., striatal PV−/− Pvalb neurons show besides increased paired-pulse facilitation, higher excitability and spontaneous spiking is more regular, likely involving changes in the activation of small conductance (SK) Ca^2+^-dependent K^+^ channels (Orduz et al., 2013).

First signs of oxidative stress evidenced by increased 8-oxo-dG and DHE signal intensity were observed at the age of 3 months in PV−/− mice. Preliminary behavioral experiments (3-chamber assay) carried out in 3 months-old male PV−/− mice indicate that the ASD-like phenotype is rather attenuated (Filice, unpublished) compared to PND25-30 mice (Filice et al., 2018) further supporting that most probably oxidative stress is not causally implicated in the development of the ASD-like phenotype of PV−/− mice. Whether this is also the case in other ASD mouse models with reduced PV levels (Supplementary Table S1 in Wöhr et al., 2015) or restricted to PV−/− mice remains to be shown.

## Data Availability Statement

All datasets generated for this study are included in the article/Supplementary Material.

## Ethics Statement

The animal study was reviewed and approved by Animal care committee (Canton of Fribourg, Switzerland); the authorization number for housing is H-04.2012-Fr and for experiments 2016_37_FR.

## Author Contributions

LJ carried out the experiments, performed data analysis, and wrote the manuscript. BS conceived the study, performed data analysis and together with LJ wrote the manuscript. All authors read and approved the final manuscript.

## Conflict of Interest

The authors declare that the research was conducted in the absence of any commercial or financial relationships that could be construed as a potential conflict of interest.
