# Supplementary material for: Parvalbumin-Deficiency Accelerates the Age-Dependent ROS Production in Pvalb Neurons in vivo: Link to Neurodevelopmental Disorders
Source: Front Cell Neurosci. 2020 Sep 28;14:571216. doi: 10.3389/fncel.2020.571216 (PMC7549402; doi:10.3389/fncel.2020.571216)
Supplement: Supplementary file 1 [file Data_Sheet_1.pdf]

Supplementary Material

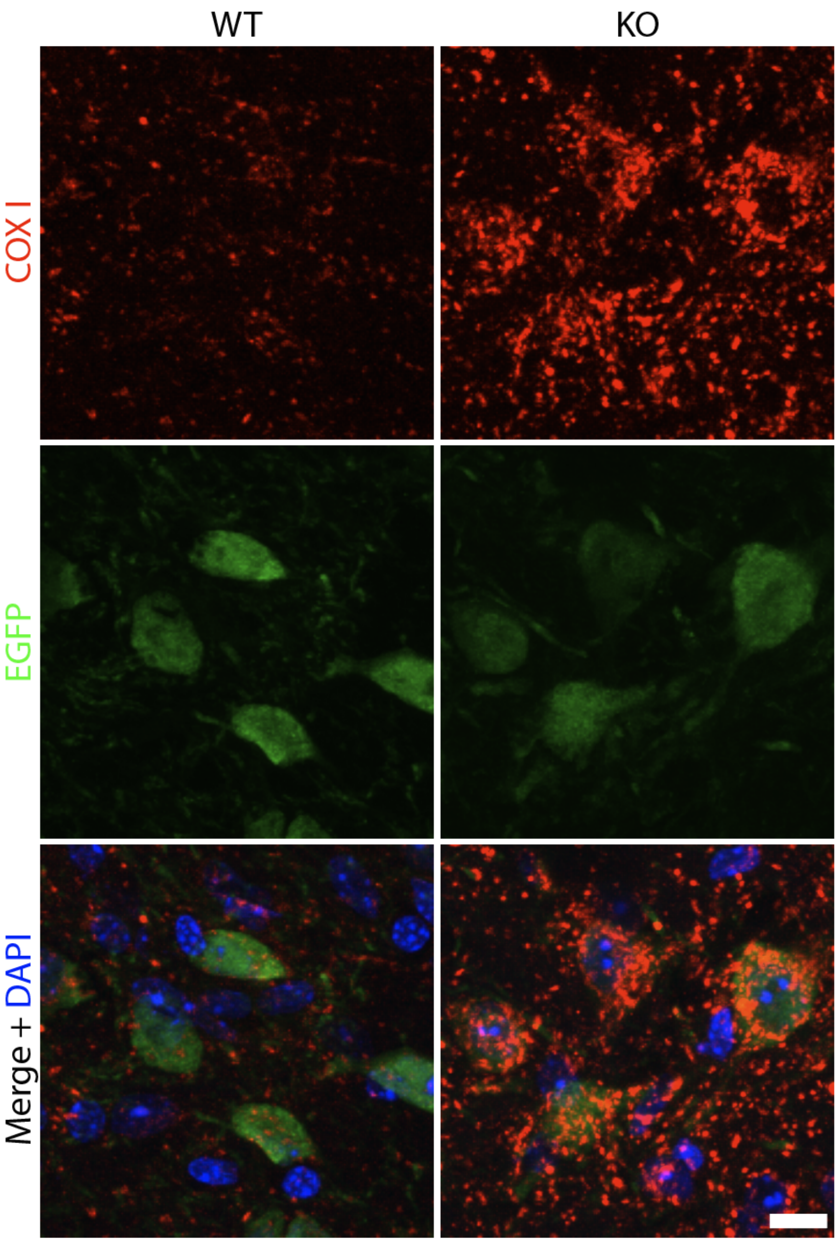

Supplementary Figure 1. The shape of Pvalb neurons is recognizable merely by a general staining for mitochondria, particularly in PV-/- (KO) mice. Pvalb neurons in the TRN were stained for COX I (red; mitochondria), EGFP (green; cytoplasm) and DAPI (blue; nucleus). The high mitochondria density in TRN Pvalb neurons of PV-/- mice allows to estimate the outline of Pvalb neurons. Scale bar: 10 µm.

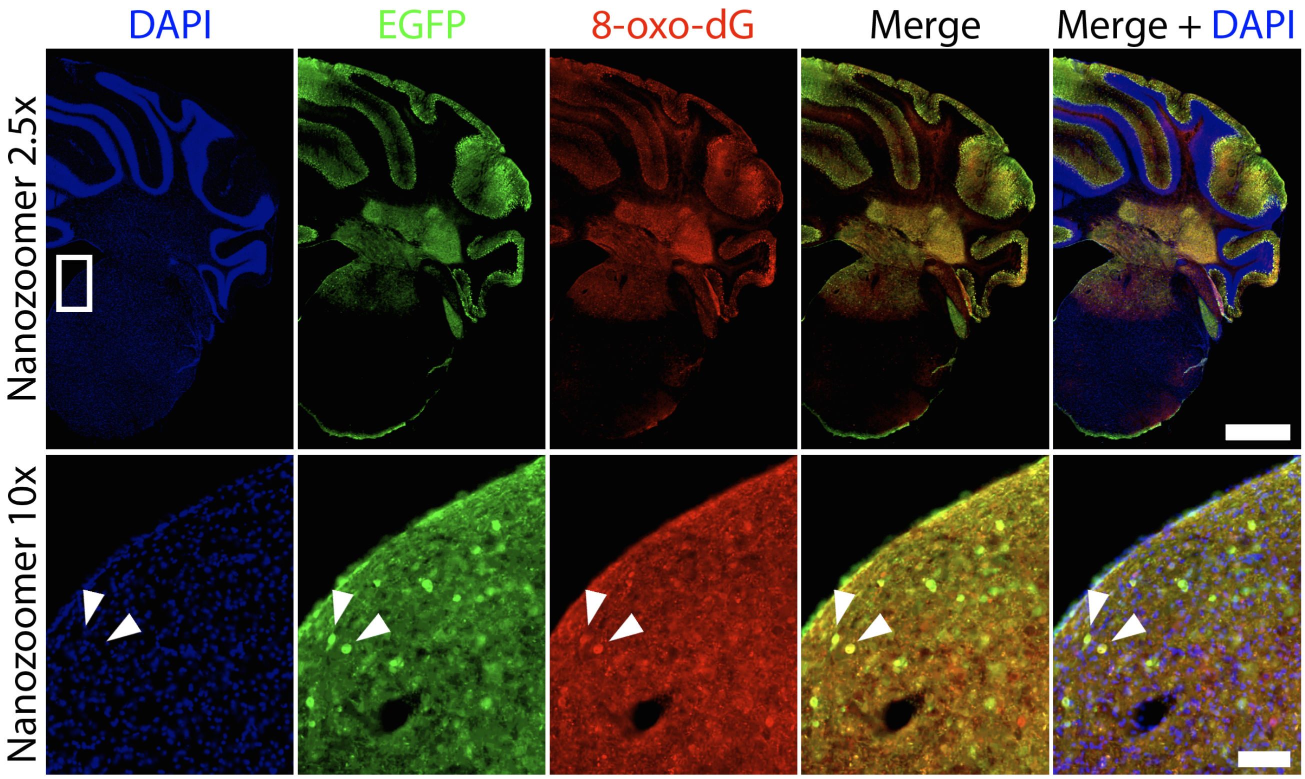

Supplementary Figure 2. Coronal brain section including medial vestibular nucleus (MVN) and cerebellum. A representative brain section scanned by the Nanozoomer of a 3-months old WT mouse is shown, stained for nuclei (DAPI, blue), EGFP (green) and 8-oxo-dG (red) at low (2.5x) magnification (upper row). A region of interest (white rectangle) shows part of the MVN at higher (10x) magnification (lower row), where few $EGFP^+$ Pvalb neurons (green) occupy the median and lower part of the parvocellular part of the medial vestibular nucleus. 8-oxo-dG staining was mostly confined to fibers and some $EGFP^+$ Pvalb neuron somata. Two neurons with strong staining for both, EGFP and 8-oxo-dG in their soma, are marked with white arrowheads. In the merged image these cells appear yellow. Scale bars upper row: 1 mm, lower row: 40 µm.

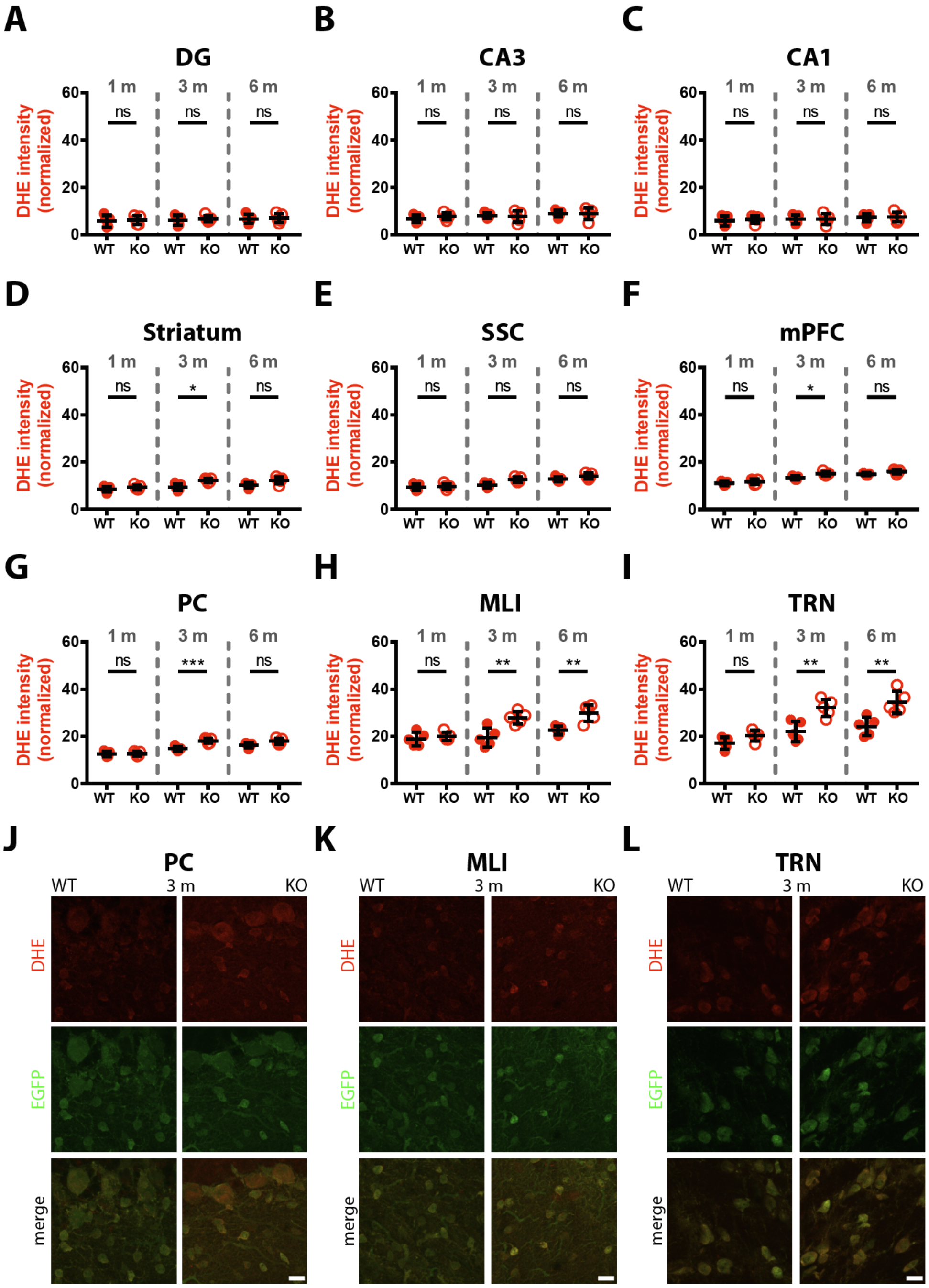

Supplementary Figure 3. Comparison of dihydroethidium (DHE) intensity between WT and KO mice at 1, 3 and 6 months. Mice at the ages of 1, 3 and 6 months were injected with DHE. Normalized ox-DHE fluorescence signal intensities (a.u.) are shown; for details on normalization, see suppl. Material in (Janickova et al., 2020). Quantitative analyses are shown for hippocampal regions DG **(A)**, CA3 **(B)**, CA1 **(C)**, striatum **(D)**, SSC **(E)**, mPFC **(F)**, Purkinje cells (PC) **(G)**, MLI **(H)** and TRN **(I)**. Each dot in the graphs represents the average obtained in 1 animal (5 mice per genotype) and 10 – 15 cells per animal resulting in >50 cells per brain region and genotype. ns - not significant; * $p<0.05$; ** $p<0.01$. Representative confocal images of 3-months old WT (left panels) and KO (right panels) mice show Purkinje cells (PC) **(J)**, MLI **(K)** and TRN Pvalb neurons **(L)**. Staining: DHE (red), EGFP (green). Scale bars: 15 µm. Note the similar results obtained by DHE staining and by 8-oxo-dG staining as shown in Figs. 2 – 4 of the main text. Values are reported in Table 1.

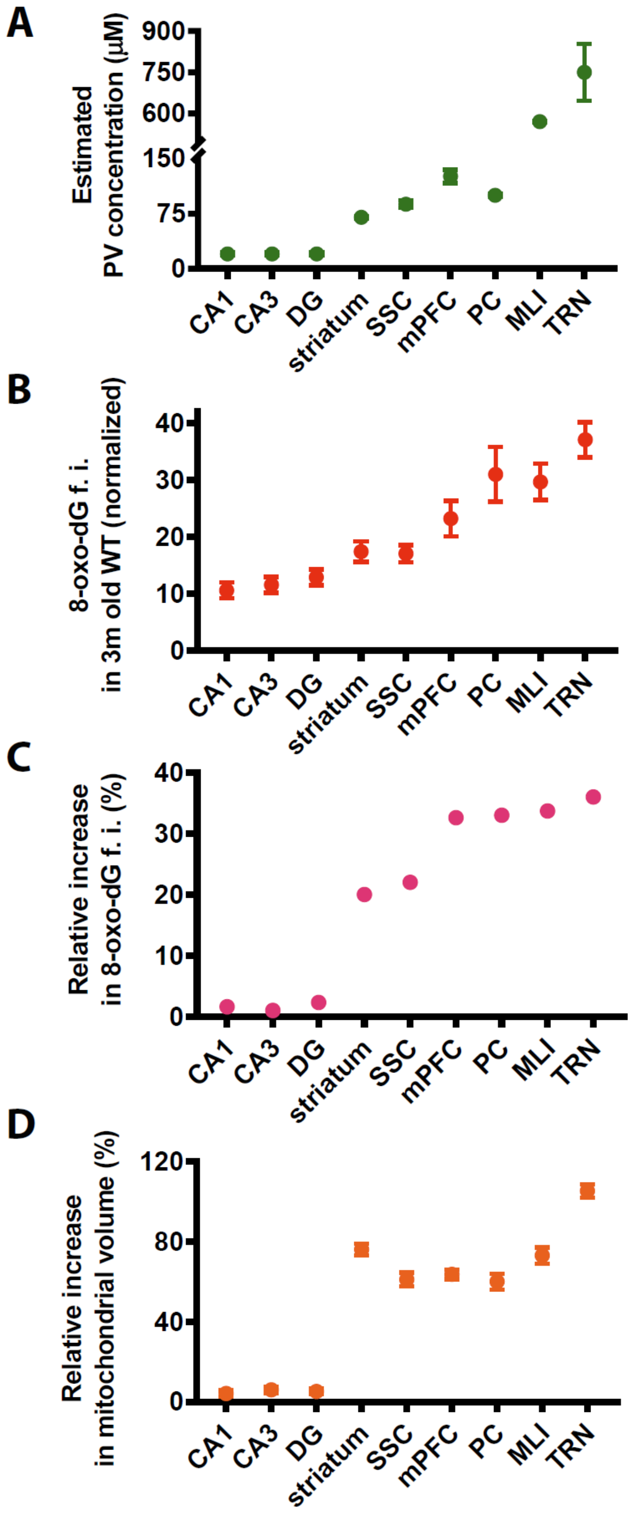

Supplementary Figure 4. Correlation between PV concentration, 8-oxo-dG fluorescence signals in WT and KO mice and PV deficiency-induced increase in mitochondria volume in Pvalb neurons. A) Estimated PV concentration in selected Pvalb neurons in 3 – 5 months-old WT mice (values taken from (Eggermann and Jonas, 2012;Janickova et al., 2020)), **B)** Normalized 8-oxo-dG fluorescence signal intensity in 3-months old WT mice strongly correlates with the PV

concentrations in WT Pvalb neurons. **C)** The relative increase in 8-oxo-dG fluorescence intensity resulting from absence of PV is generally well correlated with the PV concentration in Pvalb neurons and moreover with the relative increase in mitochondria volume caused by PV-deficiency shown in (**D**): low in hippocampus, medium in striatum and cortical regions and highest in MLI and TRN Pvalb neurons; data in **D** are taken from (Janickova et al., 2020). A divergence was observed in striatal Pvalb neurons, where the increase in mitochondria volume was over-proportional in relation to the estimated PV concentration. Yet in these Pvalb neurons, the relative increase in 8-oxo-dG fluorescence signal intensity (oxidative stress) caused by absence of PV is lower than in e.g. mPFC neurons suggesting that substantial mitochondria volume upregulation in striatal Pvalb neurons might actually decrease/delay the extent of oxidative stress. A converse situation also prevails in PC, where relative mitochondria upregulation is rather small, yet 8-oxo-dG signals are as strong as in MLI and TRN Pvalb neurons, both with considerably higher PV concentrations. However, increase in mitochondria volume in absolute terms (+ ~100 μm^3) is highest in PC. Thus, for particularly large (PC) or small (MLI) Pvalb neurons, their morphologies likely have to be taken into consideration.

References

Eggermann, E., and Jonas, P. (2012). How the 'slow' Ca(2+) buffer parvalbumin affects transmitter release in nanodomain-coupling regimes. *Nat Neurosci* 15**,** 20-22.

Janickova, L., Rechberger, K.F., Wey, L., and Schwaller, B. (2020). Absence of parvalbumin increases mitochondria volume and branching of dendrites in Pvalb neurons in vivo: a point of convergence of autism spectrum disorder (ASD) risk gene phenotypes. *Mol Autism* 11**,** 47.
